# Histone deacetylase III interactions with BK polyomavirus large tumor antigen may affect protein stability

**DOI:** 10.1186/s12985-023-02128-6

**Published:** 2023-07-18

**Authors:** Yueh-Han Hsu, Chun-Nun Chao, Hsin-Yi Huang, Pei-Wen Zhao, Pang-Hung Hsu, Cheng-Huang Shen, San-Yuan Chen, Chiung-Yao Fang

**Affiliations:** 1 Division of Nephrology, Department of Internal Medicine, Ditmanson Medical Foundation Chia-Yi Christian Hospital, Chia-Yi, Taiwan; 2 Department of Nursing, Min-Hwei Junior College of Health Care Management, Tainan, Taiwan; 3 Department of Pediatrics, Ditmanson Medical Foundation Chia-Yi Christian Hospital, Chia-Yi, Taiwan; 4 Department of Biomedical Sciences, National Chung Cheng University, Chia-Yi, Taiwan; 5 Department of Medical Laboratory Science and Biotechnology, Asia University, Taichung, Taiwan; 6 Department of Medical Research, Ditmanson Medical Foundation Chia-Yi Christian Hospital, Chia-Yi, Taiwan; 7 Department of Bioscience and Biotechnology, National Taiwan Ocean University, Keelung, Taiwan; 8 Department of Urology, Ditmanson Medical Foundation Chiayi Christian Hospital, Chia-Yi, Taiwan; 9 Department of Chinese Medicine, Ditmanson Medical Foundation Chia-Yi Christian Hospital, Chia-Yi, Taiwan; 10 Department of Sports Management, Chia Nan University of Pharmacy & Science, Tainan City, Taiwan

**Keywords:** BKPyV, Large T antigen, Acetylation, Protein stability, HDAC3

## Abstract

**Background:**

Human polyomavirus BK (BKPyV) causes associated nephropathy and contributes to urinary tract cancer development in renal transplant recipients. Large tumor antigen (LT) is an early protein essential in the polyomavirus life cycle. Protein acetylation plays a critical role in regulating protein stability, so this study investigated the acetylation of the BKPyV LT protein.

**Methods:**

The BKPyV LT nucleotide was synthesized, and the protein was expressed by transfection into permissive cells. The BKPyV LT protein was immunoprecipitated and subjected to LC-MS/MS analysis to determine the acetylation residues. The relative lysine was then mutated to arginine in the LT nucleotide and BKPyV genome to analyze the role of LT lysine acetylation in the BKPyV life cycle.

**Results:**

BKPyV LT acetylation sites were identified at Lys3 and Lys230 by mass spectrometry. HDAC3 and HDAC8 and their deacetylation activity are required for BKPyV LT expression. In addition, mutations of Lys3 and Lys230 to arginine increased LT expression, and the interaction of HDAC3 and LT was confirmed by coimmunoprecipitation.

**Conclusions:**

HDAC3 is a newly identified protein that interacts with BKPyV LT, and LT acetylation plays a vital role in the BKPyV life cycle.

**Supplementary Information:**

The online version contains supplementary material available at 10.1186/s12985-023-02128-6.

## Introduction

Human polyomavirus BK (BKPyV) is a small nonenveloped DNA virus [1, 2]. Primary BKPyV infection occurs in early childhood but is asymptomatic, with the virus remaining in a latent state in epithelial cells of the renal and urogenital tracts. When transplant patients are administered potent immunosuppressive drugs, BKPyV may reactivate, causing severe complications [3, 4], such as nephropathy and hemorrhagic cystitis in renal and bone marrow transplant recipients [5]. Moreover, recent evidence indicates that BKPyV can cause urothelial and other malignancies in transplant recipients [6–9].

The BKPyV genome encodes three early proteins, a large tumor antigen (LT), a small tumor antigen (st), and a truncated T antigen produced by alternative splicing. The LT protein of the *Polyomaviridae* family is a multifunctional protein required for viral DNA replication, late gene transcription, and viral assembly [10, 11]. By interacting with the cellular tumor suppressor proteins Rb, p107, p130, and p53, LT drives infected cells into the S phase and uncontrolled cell growth [12–14]. Additionally, BKPyV LT activates DNA methyltransferase 1 [15], possibly associated with tumor suppressor gene hypermethylation. Therefore, the BKPyV LT protein plays a pivotal role in viral life cycle completion and cellular transformation.

As obligate parasites, viruses hijack and use host machinery to complete their life cycle [16]. Since protein posttranslational modifications (PTMs) are more efficient than protein turnover in regulating protein function [17, 18], the PTM of viral proteins plays an essential role in the viral life cycle and its associated diseases [19–21]. Previously, several PTMs were found in BKPyV structural proteins and minichromosomes [22, 23]. The phosphorylation of BKPyV structural proteins is essential for viral propagation [24], but BKPyV LT protein modification has not been fully investigated. Phosphorylation of LT affects SV40 replication efficiency and interferes with host signaling pathways [25, 26]. Phosphorylation of the Merkel Cell Polyomavirus (MCPyV) LT protein impacts the initiation of viral replication [27]. The K697 residue of SV40 LT is acetylated by CBP/P300 [28], and the corresponding K697 residue of SV40 LT is conserved in BKPyV and JCPyV. Therefore, LT is suggested to be acetylated at the corresponding K697 residue in BKPyV and JCPyV [28]. Since acetylation of SV40 LT at K697 decreases protein stability [29] and histone deacetylase (HDAC)1, HDAC3, and HDAC10 may be involved in SV40 LT deacetylation, HDAC may play a positive role in polyomavirus infection. Paradoxically, inhibition of HDAC activity increases LT and structural protein expression in SV40-infected cells [30]. Additionally, treatment of JCPyV with a deacetylation inhibitor, TSA, increases JCPyV control region reporter activity [31]; thus, the role of HDAC in the polyomavirus life cycle remains unclear.

Protein acetylation normally occurs in two forms, N-terminal and lysine ε-amino group acetylation [32, 33]. Unlike N-terminal acetylation, lysine ε-amino group acetylation is reversible and regulated by the balance of histone acetyltransferases (HATs) and HDACs. To date, 18 HDACs have been identified and classified into four classes. Class I, II, and IV are Zn^2+^-dependent, whereas class III, also known as sirtuins, uses NAD^+^ as a cosubstrate for its deacetylase activity [32]. Class I HDACs, including HDAC1, HDAC2, HDAC3, and HDAC8, are the most studied and have been reported to play a critical role in the viral life cycle and associated diseases [34]. In the current study, we identified BKPyV LT acetylated residues by mass spectrometry and found that HDAC3 interacted with BKPyV LT, which may affect LT protein stability.

## Materials and methods

### Cell lines and cell culture

Human kidney, proximal tubular cells (HK-2 cells), and Vero cells were obtained from the Bioresource Collection and Research Center, Taiwan. HK-2 cells were cultured in keratinocyte-serum medium (Gibco, Life Technologies) supplemented with 5 ng/ml recombinant epidermal growth factor and 50 μg/ml bovine pituitary extract (Gibco). Vero cells were cultured in minimum essential medium with the addition of nonessential amino acids, sodium pyruvate, fetal bovine serum, and penicillin/streptomycin antibiotic solution (Gibco). These cell lines were tested for the absence of mycoplasma contamination and incubated in a humidified atmosphere containing 5% CO_2_ at 37 °C.

### Propagation and purification of BKPyV

BKPyV was propagated and purified as previously described [22, 23]. Briefly, BKPyV (UT strain) was propagated in Vero cells, which were lysed by three 60 min cycles of freezing and thawing at -80 °C and 37 °C. The viruses were released by incubation with type V neuraminidase (Sigma, St. Louis, MO), and the BKPyV-containing supernatant was purified using a 20% sucrose cushion and CsCl gradient centrifugation.

### Chemicals, reagents, and antibodies

HDAC inhibitors, tacedinaline (CI994) for HDAC1, RGFP966 for HDAC3, and PCI-34,051 for HDAC were purchased from Selleck Chemicals (Houston, TX, USA). The proteins were extracted with a NE-PER cytoplasmic extraction reagent kit (Thermo Fisher Scientific, Waltham, Massachusetts, USA) for western blotting. The protease inhibitor was purchased from Millipore (Billerica, Massachusetts, USA). SV40 LT mouse monoclonal antibody, which cross-reacts with BKPyV LT, was purchased from Millipore. Primary antibodies against HDAC1, HDAC2, HDAC3, and HDAC8 were purchased from Cell Signaling Technology Inc. (Danvers, Massachusetts, USA). GAPDH expression was used as an internal control for western blotting, and the GAPDH antibody (monoclonal, rabbit anti-human GAPDH) was purchased from Cell Signaling Technology Inc.

### Identification of BKPyV LT acetylation

The experimental workflow is shown in Fig. S1. Briefly, complete BKPyV (Accession No. DQ305492) LT gene nucleotide sequences were custom synthesized and optimized by GenScrip (GenScript Biotech, Inc., NJ, USA) without altering the amino acid sequences. The synthesized LT complete nucleotide was then cloned into the HindIII/EcoRI restriction site of the pcDNA3.1 plasmid with the C-terminus fused with a Myc tag (GenScript Biotech) to generate the BKPyV LT-C-Myc/pcDNA3.1 plasmid. The plasmid was then transfected into HK-2 cells for BKPyV LT protein expression. The LT protein was purified by immunoprecipitation and subjected to SDS-PAGE and Coomassie blue staining. The band corresponding to the LT protein was excised for mass spectrometric analysis.

### Immunoprecipitation

Aliquots (10 μg) of LT antibody (Millipore) were added to 50 μL of magnetic Dynabeads M-280 and incubated for 12 min by rotation on a roller at 25 °C. The antibody-conjugated beads were washed three times with PBS containing Tween 20 (0.025%). Forty micrograms of separated nuclear lysates were incubated with antibody-conjugated beads for 12 min by rotation on a roller at 25 °C and then washed three times with PBS/Tween 20 before the bound LT protein was subjected to SDS-PAGE and western blotting analysis.

### In-gel digestion

The excised gel band was first destained and then reduced with 10 mM DTT (Merck, Darmstadt, Germany) at 56 °C for 45 min, followed by cysteine blocking with 55 mM iodoacetamide (IAM, Sigma, St. Louis, MO, USA) at 25 °C for 30 min. Samples were digested with sequencing-grade modified porcine trypsin (Promega, Madison, WI, USA) at 37 °C for 16 h. The peptides were then extracted from the gel, dried by vacuum centrifugation, and stored at -80 °C until use.

### LC-MS/MS analysis

The digested peptide was reconstituted in HPLC buffer A (0.1% formic acid) and loaded onto a reverse-phase column (Zorbax 300SB-C18, 0.3 × 5 mm; Agilent Technologies, Wilmington, DE, USA). The desalted peptides were then separated on a homemade column (HydroRP 2.5 μm, 75 μm ID x 20 cm with a 15 μm tip) using a multistep gradient of HPLC buffer B (99.9% acetonitrile/0.1% formic acid) for 60 min with a flow rate of 0.25 μl/min. The LC apparatus was coupled with a 2D linear ion trap mass spectrometer (Orbitrap Elite ETD; Thermo Fisher, San Jose, CA, USA) operated using Xcalibur 2.2 software (Thermo Fisher). Full-scan MS was performed in the Orbitrap over a range of 400 to 2,000 Da and a resolution of 120,000 at m/z 400. Internal calibration was performed using the ion signal of [Si(CH3)_2_O]6 H + at m/z 536.165365 as the lock mass. The 20 data-dependent MS/MS scan events were followed by one MS scan for the 20 most abundant precursor ions in the preview MS scan. The m/z values selected for MS/MS were dynamically excluded for 40 s with a relative mass window of 15 ppm. The electrospray voltage was set to 2.0 kV, and the temperature of the capillary was set to 200 °C. MS and MS/MS automatic gain control were set to 1,000 ms (full scan) and 200 ms (MS/MS) or 3 × 10^6^ ions (full scan) and 3 × 10^3^ ions (MS/MS) for maximum accumulated time or ions, respectively.

### Protein identification

The data were analyzed using Proteome Discoverer software (version 1.4, Thermo Fisher Scientific), and the MS/MS spectra were searched in the UniProt database (downloaded on May 15, 2018; extracted for *Homo sapiens*, 20,341 sequences; and LT protein of BK polyomavirus, 1 sequence) using the Mascot search engine (Matrix Science, London, UK; version 2.5). For peptide identification, 10 ppm mass tolerance was permitted for intact peptide masses, and 0.5 Da for CID fragment ions with allowance for three missed cleavages made from the trypsin or semitrypsin digestion: oxidized methionine, oxidized proline, oxidized lysine, and acetyl (protein N-terminal) as variable modifications; carbamidomethyl (cysteine) as a static modification. Peptide-spectrum match (PSM) was then filtered based on high confidence and Mascot search engine rank 1 of peptide identification to ensure an overall false discovery rate below 0.01. Proteins with a single peptide hit were removed.

### Construction of K3R, K230R, and K3R/K230R mutation BKPyV LT plasmids

The lysine residues Lys(K)3 and K230 in BKPyV LT were found to be acetylated in the mass spectrometric analysis. To analyze K3 and K230R acetylation function in the LT protein, aag in nucleotide (nt) 7 to 9 and nt 688 to 690 were mutated to cgg to generate the K3R and K230R LT proteins, respectively. The mutated nucleotide was confirmed by sequencing, and the relative amino acid is shown in Fig. S2.

### RNA silencing

Double-stranded siRNA oligomers were purchased from Ambion and transfected using Lipofectamine 3000 (Invitrogen) according to the manufacturer’s protocol. Two siRNAs targeting sequences relative to mRNA were used for efficient silencing. Briefly, cells were grown to 80% confluence for 24 h and transfected with specific siRNA. The RNA sequence for silencing the target protein is shown in Table S1, and specific silencing was validated by western blotting to determine the relative protein expression.

### Immunofluorescence assay

HK-2 cells were seeded on a coverglass, and after transfection or virus infection, cells were fixed and permeabilized with cold acetone and methanol at a 2:1 volume ratio. The fixed cells were blocked with normal horse serum and incubated with mouse monoclonal antibody against SV40 LT (Millipore) at 4 °C overnight. Alexa 488-conjugated goat anti-mouse IgG was used as a secondary antibody (Molecular Probes). Stained coverslips were mounted with anti-fade fluorescence mounting medium (Sigma), and 1,000 cells per sample were counted using a fluorescence microscope (Olympus) to determine the percentage of LT-positive cells. Cell morphology was visualized by Evans Blue staining (Sigma).

### Intracellular staining of LT protein for flow cytometric analysis

Intracellular LT protein staining was performed by using a BD Cytofix/Cytoperm™ Fixation/Permeabilization kit (BD Biosciences, San Diego, CA). After transfection or virus infection, HK-2 cells were washed and fixed with BD Fixation/Permeabilization solution for 20 min at 4 °C. After washing with BD Perm/Wash buffer, cells were incubated with SV40 LT antibody (Millipore, cross-reaction with BKPyV LT) for 60 min at 4 °C. Mouse IgG2a, κ Isotype Ctrl Antibody (BioLegend, San Diego, California, USA) was used as an isotype control for SV40 LT antibody staining. After washing with BD Perm/Wash buffer, the cells were further incubated with Alexa 488-conjugated goat anti-mouse IgG secondary antibody (Molecular Probes) for 30 min at 4 °C. Samples were finally washed with BD Perm/Wash buffer and resuspended in PBS for flow cytometric analysis (FACSDiva; BD Biosciences).

### Cell nuclear-cytoplasmic fractionation

Nuclear extraction was conducted using a NE-PER cytoplasmic extraction reagent kit (Thermo Fisher Scientific) according to the manufacturer’s instructions. Briefly, the cytoplasmic fraction was extracted with cytoplasmic extraction buffers I and II by vortexing. The nuclear fraction was extracted in nuclear extraction buffer, and the protein was quantified using Bio-Rad Protein Assay reagent (Bio-Rad, Hercules, CA, USA).

### Construction of acetylation-dead LT mutation in the BKPyV genome to obtain mutant BKPyV

Previously, the whole BKPyV genome was cloned into a T-easy vector at the *Pst*I site. The LT sequence was mutated to generate acetylation-dead LT in the BKPyV genome. The primer pairs used to create the K3R/K230R LT BKPyV mutant are shown in Supplementary Table 1. Wild-type LT BKPyV or K3R/K230R LT BKPyV was digested by *Pst* I, and the complete BKPyV genomic DNA was then transfected into HK-2 cells. Three days posttransfection, cells were harvested to determine wild-type or K3R/K230R BKPyV LT expression.

### Western blotting

Forty micrograms of total protein or nuclear protein was subjected to SDS-PAGE and electrotransferred onto a PVDF membrane (Bio-Rad, Hercules, CA, USA). The membrane was blocked by incubation with 5% bovine serum albumin (Sigma-Aldrich) at 25 °C for 1 h, followed by probing with the primary specific antibody at 4 °C for 16 h. The membrane was then incubated with horseradish peroxidase-conjugated light chain specific secondary antibody (Jackson ImmunoResearch Laboratories, West Grove, Pennsylvania, USA) for 2 h at 25 °C. Protein expression was detected using an enhanced chemiluminescence horseradish peroxidase substrate detection kit (WBKLS0500, Millipore) and quantified using the UVP BioSpectrum 800 Imaging System (Cambridge, UK).

### Statistical analysis

Statistical analysis was performed using GraphPad Prism 7.0 (GraphPad Software, San Diego, California, USA). Data were analyzed using one-way analysis of variance. All in vitro experiments were repeated three times and presented as the mean ± SD. A *P*-value less than or equal to 0.05 was considered statistically significant and indicated by *. **: *P*-value between 0.001 and 0.01. ***: *P*-value between 0.0001 and 0.001.

## Results

### The K3 and K230 acetylation sites in BKPyV LT may affect protein stability

**Fig. 1:**
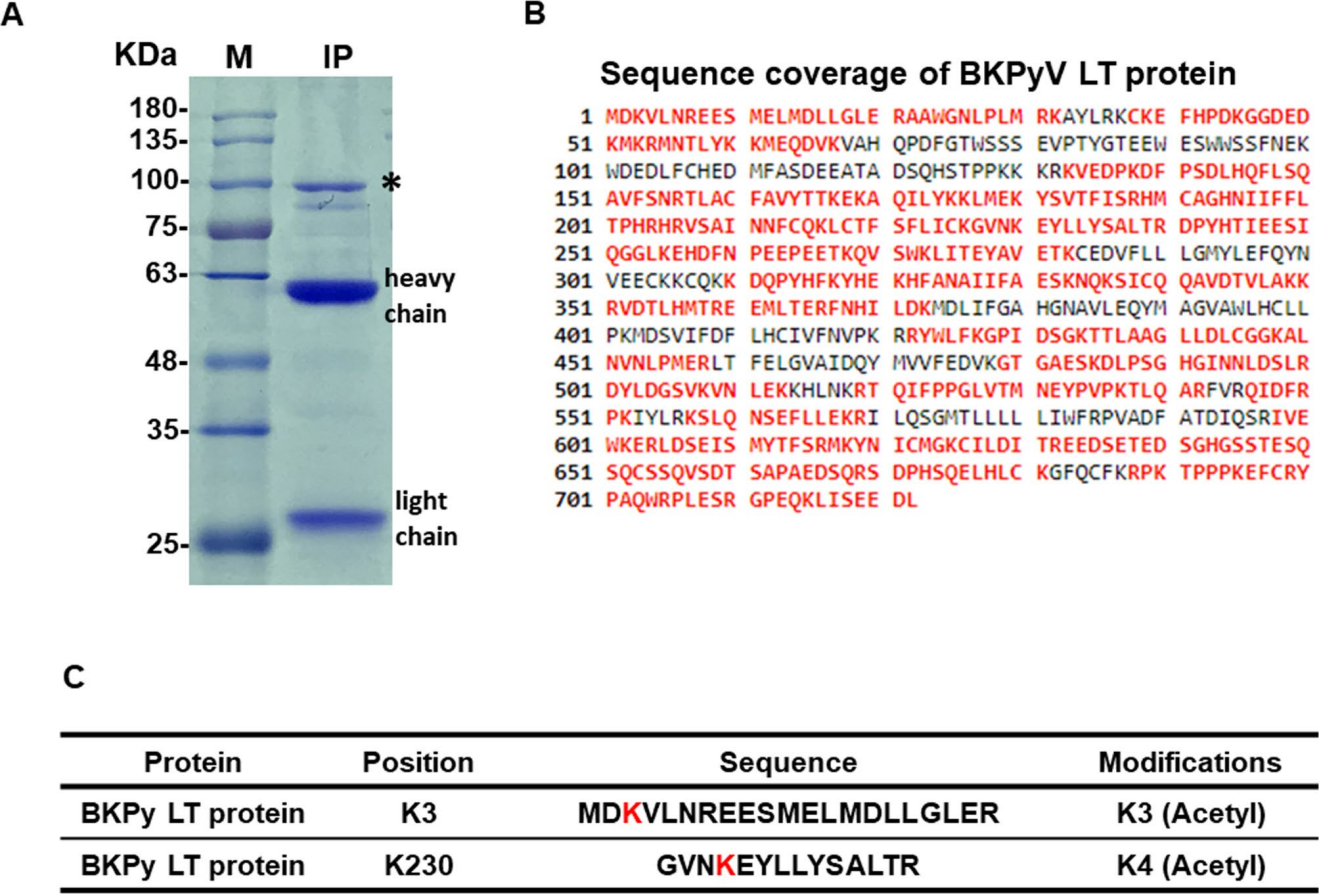
Acetylated residues in BKPyV LT were determined by mass spectrometry. **A** Immunoprecipitation of BKPyV LT protein. LT protein was transfected into HK-2 cells, and transfected cells were subjected to nuclear/cytoplasmic fractionation 48 h later. Nuclear protein was immunoprecipitated using an anti-LT antibody and analyzed by SDS-PAGE followed by Coomassie blue staining. The band corresponding to LT protein was excised and in-gel digested by trypsin. **B** Sequence coverage of the BKPyV LT protein, indicated in red. **C** K3 and K230 residues of BKPyV LT were acetylated. Digested peptides were analyzed by mass spectrometry, and the identified acetylated lysine residues are marked in red (*: The immunoprecipitated LT protein; M: protein molecular weight marker; IP: immunoprecipitation)

**Fig. 2:**
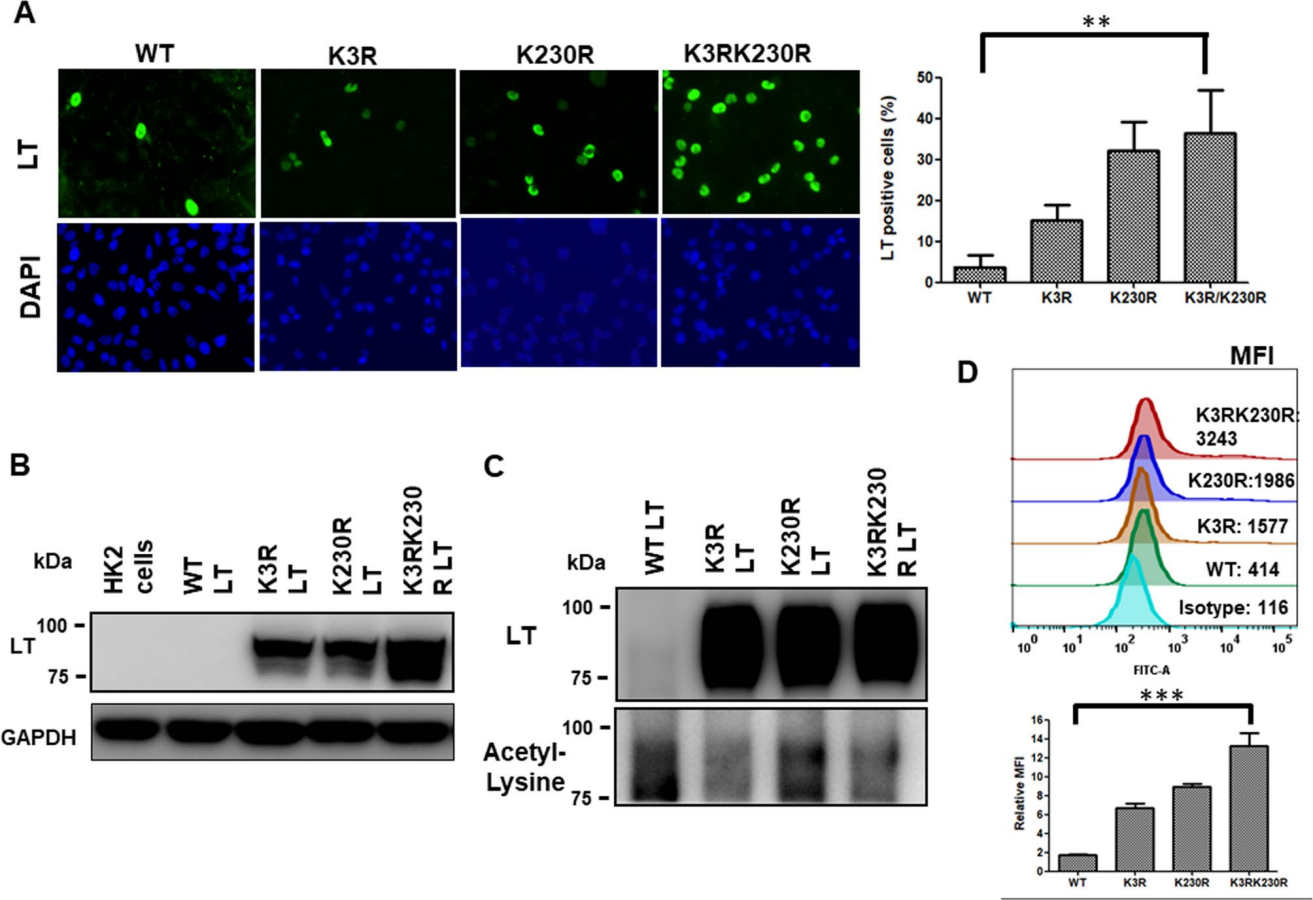
BKPyV LT expression increased when both K3 and K230 residues were mutated to arginine. K3 or K230 in LT was single or both mutated to arginine and labeled K3R, K230R, and K3R/K230R LT. **A** Wild-type (WT) and mutated LT were transfected into HK-2 cells before LT protein expression was determined 48 h posttransfection. **B** WT and mutated LT were transfected into HK-2 cells, and LT protein expression was determined by western blotting 48 h posttransfection. **C** WT and mutated LT were transfected into HK-2 cells, and the nuclear/cytoplasmic fraction was separated 48 h posttransfection. LT protein was immunoprecipitated and detected by the acetyl-lysine antibody. **D** WT and mutated LT DNA were transfected into HK-2 cells, and LT protein expression was determined by flow cytometric analysis 48 h posttransfection. Data are representative of at least three independent experiments and are shown as the mean ± SD (* *P* < 0.05; ** *P* < 0.01; *** *P* < 0.001). (MFI: Mean fluorescence intensity)

Mass spectrometry analysis revealed potential BKPyV LT acetylated lysine (K) residues K3 and K230 (Fig. 1). Then, the corresponding lysine residues (nucleotide: aag) were mutated into arginine (nucleotide: cgg) to generate pseudodeacetylated mimic (K3R, K230R, and K3R/K230R) forms of BKPyV LT proteins (Fig. S2) to determine how these two lysine acetylations modulated LT protein expression. The immunofluorescent assay showed that when both K3 and K230 lysine residues in LT were mutated to arginine, the number of LT-positive cells significantly increased (Fig. 2A). This was confirmed by transfecting K3R, K230R, and K3R/K230R LT plasmid DNA into HK-2 cells, as K3R/K230R LT expression increased compared to the single K3R or K230R mutation LT, but wild-type LT was undetectable by western blotting (Fig. 2B). It was hypothesized that the acetylation status of the LT protein might affect its expression, so the wild-type and the three pseudodeacetylation mimics (K3R, K230R, and K3R/K230R) of LT were immunoprecipitated after transfection into HK-2 cells. The western blotting results showed that after the mutation of K3 and K230 to arginine in BKPyV LT, the acetylation level decreased, and the expression increased (Fig. 2C). Quantification of LT protein expression by flow cytometry showed similar results (Fig. 2D). These data suggest that acetylation of K3 and K230 in BKPyV LT may play an essential role in regulating LT protein stability.

### HDAC3 and HDAC8 are required for BKPyV LT protein expression

**Fig. 3:**
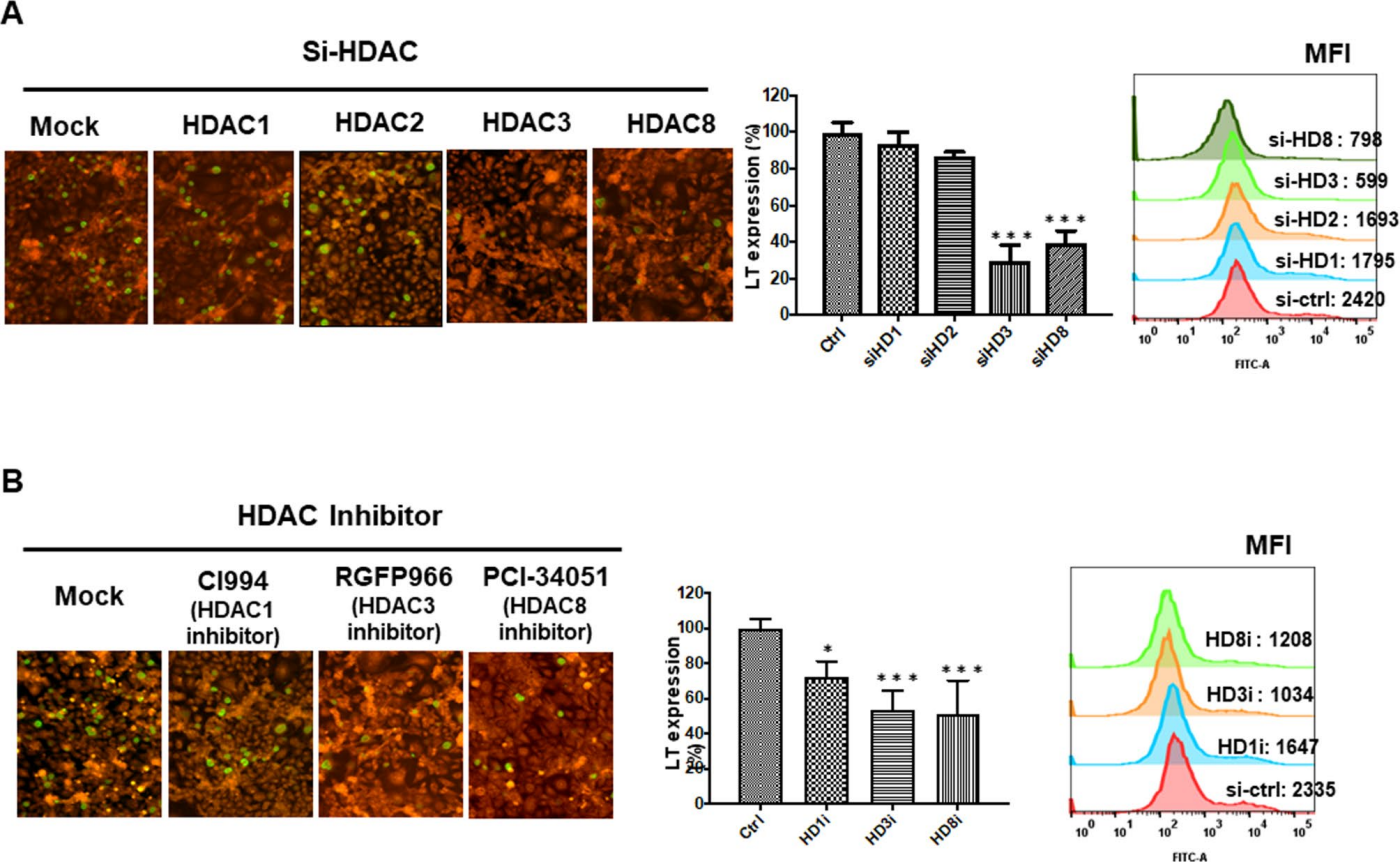
HDAC3 and HDAC8 are required for BKPyV LT protein expression. **A** The expression of class I HDACs was knocked down by siRNA, then cells were infected with BKPyV and LT protein expression was determined by immunofluorescence and flow cytometric analysis 48 h post-infection. Middle panel: Quantification of the LT expression levels in the immunofluorescence assay. Right panel: LT protein expression was determined by intracellular staining and quantified by flow cytometry. **B** Class I HDACs were inhibited by HDAC inhibitors, cells were infected with BKPyV, and LT protein expression was determined by immunofluorescence and flow cytometric analysis 48 h postinfection. Middle panel: Quantification of the LT expression levels in the immunofluorescence assay. Right panel: LT protein expression was determined by intracellular staining and quantified by flow cytometry. BKPyV infection without siRNA or HDAC inhibitor treatment was considered 100%. (Green, LT protein; Red, Evan’s Blue stain). Data are representative of at least three independent experiments and are shown as the mean ± SD (* *P* < 0.05; ** *P* < 0.01; *** *P* < 0.001). (MFI: Mean fluorescence intensity)

HAT and HDACs control lysine ε-amino group acetylation in nonhistone proteins [35], so they may play a role in the BKPyV life cycle. HDAC1, HDAC2, HDAC3, and HDAC8 expression was silenced using specific siRNA in HK-2 cells (Fig. S3) before the cells were infected with BKPyV to determine early LT protein expression. BKPyV LT expression decreased to approximately 30% or 40% when HDAC3 or HDAC8 was knocked down, respectively (Fig. 3A), indicating that they are required for BKPyV infection. The use of the HDAC inhibitors PGFP966 for HDAC3 and PCI-34,051 for HDAC8 reduced the ratio of BKPyV LT-positive cells to approximately 50% in HDAC3 and HDAC8 inhibitor-treated cells (Fig. 3B). Quantification of LT by flow cytometric analysis showed similar results. The data indicate that HDAC3 and HDAC8 and their deacetylation activity are vital for the BKPyV life cycle.

### HDAC3 interacts with BKPyV LT protein

**Fig. 4:**
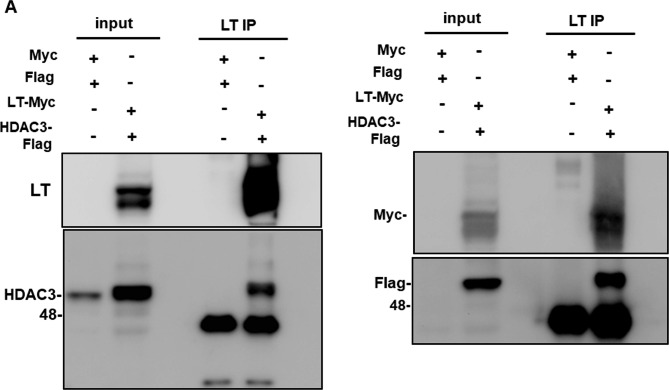
HDAC3 interacts with BKPyV LT. **A** LT fused with a Myc tag (LT_myc) and HDAC3 fused with a flag tag (HDAC3_Flag) were cotransfected into HK-2 cells. Forty-eight hours later, transfected cells were immunoprecipitated with LT antibody to detect the presence of HDAC3 by western blotting. **B** LT fused with a Myc tag (LT_myc) and HDAC3 fused with a flag tag (HDAC3_Flag) were cotransfected into HK-2 cells. Forty-eight hours later, transfected cells were immunoprecipitated with LT antibodies to detect the LT-myc and HDAC3_Flag fusion proteins

The possible interaction of HDAC3 or HDAC8 with BKPyV LT was analyzed by coimmunoprecipitation. BKPyV LT fused with a Myc tag was cotransfected with HDAC3 or HDAC8 fused with a flag tag into HK-2 cells. Unfortunately, HDAC8 protein expression was undetectable (data not shown), but HDAC3 expression was detected in LT-immunoprecipitated HK-2 cells (Fig. 4A), indicating that HDAC3 interacts with the BKPyV LT protein. This interaction was confirmed using a fusion tag, Myc antibody (presentative of LT), or flag tag antibody (presentative of HDAC3), as the Myc tag was detected in LT-Myc transfected HK-2 cells and flag tag in LT immunoprecipitated cells at the molecular weight corresponding to HDAC3 (Fig. 4B). Taken together, these data suggest that the BKPyV LT protein interacts with HDAC3.

### Role of LT K3 and K230 acetylation in BKPyV life cycle LT expression

**Fig. 5:**
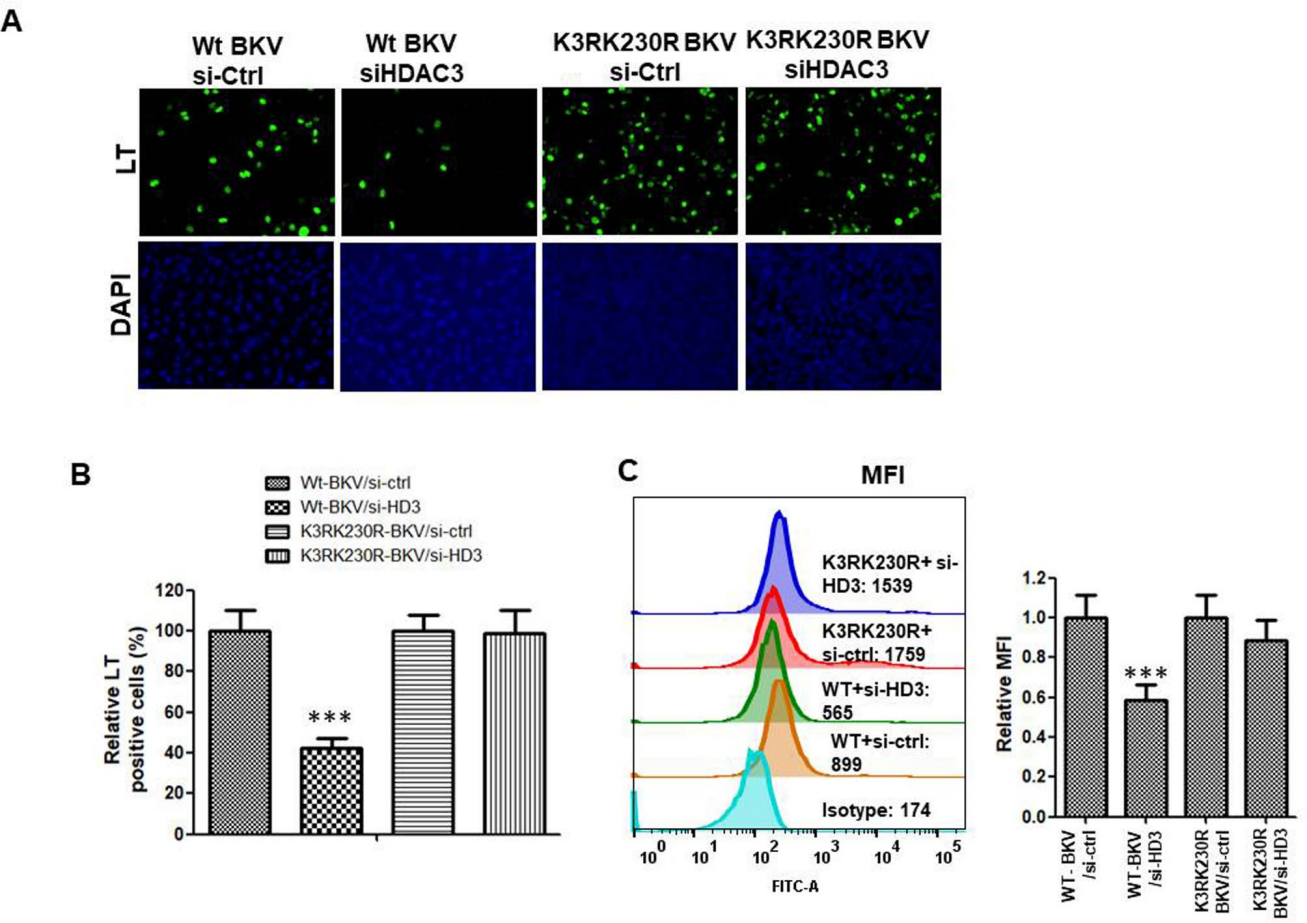
HDAC3 is critical in the WT BKPyV life cycle. Endogenous HDAC3 was knocked down by siRNA for 24 h, and the wild-type or K3R/K230R BKPyV genome was transfected into HK-2 cells. Seventy-two hours later, cells were harvested for immunofluorescent assay to determine LT protein expression. **A** BKpyV LT expression was determined by immunofluorescence. **B** Quantification of the LT expression ratio. **C** LT protein expression was quantified by flow cytometric analysis. Data are representative of at least three independent experiments and are shown as the mean ± SD (* *P* < 0.05; ** *P* < 0.01; ns, not significant). (MFI: Mean fluorescence intensity)

The K3R/K230R LT mutant BKPyV was constructed to further analyze the role of K3 and K230 acetylation of LT protein expression in the BKPyV life cycle. Endogenous HDAC3 in HK-2 cells was knocked down by siRNA, then transfected with the linear wild-type and K3R/K230R mutated BKPyV genome. Seventy-two hours later, LT protein expression was detected by immunofluorescence or quantified by flow cytometric analysis, and as expected, LT expression in the K3R/K230R LT mutant BKPyV was not significantly affected when HDAC3 was knocked down, whereas LT expression in wild-type BKPyV decreased to approximately 40% (Fig. 5). These data suggest that HDAC3 is critical for LT K3 and K230 deacetylation and essential in BKPyV infection.

## Discussion

Since proteomics is a powerful tool for analyzing protein posttranslational modification [36] and nonhistone protein acetylation is involved in vital processes related to physiology and disease progression [35, 37], BKPyV LT acetylation was explored by a proteomics approach, revealing that K3 and K230 are acetylated. Comparing the structure domain with SV40, the acetylated K3 and K230 of BKPyV LT are located within the J and origin binding domains (OBD), respectively [10]. The J domain is responsible for Hsc70 and Rb protein binding, and the OBD is responsible for Zn binding. Since neither K3 nor K230 is at the binding motif of Hsc70 and Rb, further investigation is required to determine whether acetylated K3 or K230 affects the function of these domains.

Lysine residues are frequently substituted with arginine to mimic non-acetylated lysine by preserving the positive charge from acetylation [38]. In the current study, K3 and K230 were mutated to arginine to analyze BKPyV LT expression. However, there is a difference in the overall structure using arginine instead of lysine [38, 39], so there may be some limitations in the application of this mutation method. When K3 and K230 in LT were mutated to arginine, the LT protein expression level increased, which may be related to LT protein stability. Lysine acetylation may regulate protein degradation [40]. For example, acetylation and ubiquitination compete with identical lysine residues to regulate SMAD7 protein proteosome-dependent degradation [41]. Neither K3 nor K230 ubiquitination was found in our mass spectrometric study. Previously, K697 acetylation was reported to regulate SV40 LT protein stability [29]. Based on sequence homology, K687 in BKPyV LT corresponds to K697 in SV40 LT [28]. Peptides containing K687 were not sequenced in our mass spectrometric analysis, so whether K687 posttranslational modification regulates BKPyV LT stability needs further investigation. We also found that when only WT LT DNA was transfected into cells, the LT protein was relatively unstable when compared with the transfection of the whole BKV genome (Figs. 2 and 5). It seemed that other unidentified mechanisms played a role in stabilizing the LT protein in the BKPyV life cycle.

Pan-HDAC inhibitors can enhance human polyomavirus JCPyV transcription [31], and HDACs play a negative role in the JCPyV life cycle. Previously, HDAC1, HDAC3, and HDAC10 were shown to interact with SV40 LT protein in COS-7 cells [29]. HDAC1, HDAC3, and HDAC10 increase constitutive SV40 LT protein stability by deacetylating the K697 residue. In the present study, HDAC3 and HDAC8 and their deacetylase were essential for BKPyV infection, and HDAC3 interacted with BKPyV LT. HDAC3 regulates non-histone protein stability by deacetylation, such as cyclin A [42] and the regulator of calcineurin 1 [43]. According to the present study, HDAC3 knocked down or repressed its deacetylase activity by specific inhibitors that suppressed BKPyV LT protein expression; thus, HDAC3 might regulate LT protein stability.

In summary, the BKPyV LT protein is acetylated at the K3 and K230 residues, which may affect its stability. HDAC3 is an unreported BKPyV LT interaction protein vital for BKPyV infection. Our results reveal the critical role of HDACs in the BKPyV life cycle, possibly by stabilizing viral oncoprotein expression.

## Electronic supplementary material

Below is the link to the electronic supplementary material.

Supplementary Material 1

## Data Availability

All data and materials described in this study are included in this manuscript and supplementary files.

## List of abbreviations

BKPyV: Human polyomavirus BK
HDAC: Histone deacetylase
K: Lysine
LC-MS/MS: Liquid chromatography-tandem mass spectrometry
LT: Larger tumor antigen
MFI: Mean fluorescence intensity
PTM: Posttranslational modification
R: Arginine

## References

[1] Helle F, Brochot E, Handala L, Martin E, Castelain S, Francois C, Duverlie G. Biology of the BKPyV: an update. Viruses 2017, 9. 10.3390/v9110327 PMC5707534 29099746

[2] Ambalathingal GR, Francis RS, Smyth MJ, Smith C, Khanna R (2017). BK Polyomavirus: clinical aspects, Immune Regulation, and emerging therapies. Clin Microbiol Rev.

[3] Nickeleit V, Singh HK (2015). Polyomaviruses and disease: is there more to know than viremia and viruria?. Curr Opin Organ Transplant.

[4] Kuypers DR (2012). Management of polyomavirus-associated nephropathy in renal transplant recipients. Nat Rev Nephrol.

[5] Chong S, Antoni M, Macdonald A, Reeves M, Harber M, Magee CN (2019). BK virus: current understanding of pathogenicity and clinical disease in transplantation. Rev Med Virol.

[6] Papadimitriou JC, Randhawa P, Rinaldo CH, Drachenberg CB, Alexiev B, Hirsch HH (2016). BK Polyomavirus infection and Renourinary Tumorigenesis. Am J Transplant.

[7] Starrett GJ, Buck CB (2019). The case for BK polyomavirus as a cause of bladder cancer. Curr Opin Virol.

[8] Dufek S, Haitel A, Muller-Sacherer T, Aufricht C (2013). Duct Bellini carcinoma in association with BK virus nephropathy after lung transplantation. J Heart Lung Transplant.

[9] Sirohi D, Vaske C, Sanborn Z, Smith SC, Don MD, Lindsey KG, Federman S, Vankalakunti M, Koo J, Bose S (2018). Polyoma virus-associated carcinomas of the urologic tract: a clinicopathologic and molecular study. Mod Pathol.

[10] An P, Saenz Robles MT, Pipas JM (2012). Large T antigens of polyomaviruses: amazing molecular machines. Annu Rev Microbiol.

[11] Topalis D, Andrei G, Snoeck R (2013). The large tumor antigen: a “Swiss Army knife” protein possessing the functions required for the polyomavirus life cycle. Antiviral Res.

[12] Harris KF, Christensen JB, Imperiale MJ (1996). BK virus large T antigen: interactions with the retinoblastoma family of tumor suppressor proteins and effects on cellular growth control. J Virol.

[13] Shivakumar CV, Das GC (1996). Interaction of human polyomavirus BK with the tumor-suppressor protein p53. Oncogene.

[14] Harris KF, Christensen JB, Radany EH, Imperiale MJ (1998). Novel mechanisms of E2F induction by BK virus large-T antigen: requirement of both the pRb-binding and the J domains. Mol Cell Biol.

[15] McCabe MT, Low JA, Imperiale MJ, Day ML (2006). Human polyomavirus BKV transcriptionally activates DNA methyltransferase 1 through the pRb/E2F pathway. Oncogene.

[16] Spriggs CC, Harwood MC, Tsai B (2019). How non-enveloped viruses hijack host machineries to cause infection. Adv Virus Res.

[17] Millar AH, Heazlewood JL, Giglione C, Holdsworth MJ, Bachmair A, Schulze WX (2019). The scope, functions, and Dynamics of Posttranslational protein modifications. Annu Rev Plant Biol.

[18] Walsh CT, Garneau-Tsodikova S, Gatto GJ (2005). Protein posttranslational modifications: the chemistry of proteome diversifications. Angew Chem Int Ed Engl.

[19] Kfoury Y, Nasr R, Journo C, Mahieux R, Pique C, Bazarbachi A (2012). The multifaceted oncoprotein tax: subcellular localization, posttranslational modifications, and NF-kappaB activation. Adv Cancer Res.

[20] Giese S, Ciminski K, Bolte H, Moreira EA, Lakdawala S, Hu Z, David Q, Kolesnikova L, Gotz V, Zhao Y (2017). Role of influenza a virus NP acetylation on viral growth and replication. Nat Commun.

[21] Thomas Y, Androphy EJ. Acetylation of E2 by P300 mediates topoisomerase entry at the Papillomavirus Replicon. J Virol 2019, 93. 10.1128/JVI.02224-18 PMC6430547 30651357

[22] Fang CY, Chen HY, Wang M, Chen PL, Chang CF, Chen LS, Shen CH, Ou WC, Tsai MD, Hsu PH, Chang D (2010). Global analysis of modifications of the human BK virus structural proteins by LC-MS/MS. Virology.

[23] Fang CY, Shen CH, Wang M, Chen PL, Chan MW, Hsu PH, Chang D (2015). Global profiling of histone modifications in the polyomavirus BK virion minichromosome. Virology.

[24] Chen PL, Hsu PH, Fang CY, Chang CF, Ou WC, Wang M, Chang D (2011). Phosphorylation of Ser-80 of VP1 and Ser-254 of VP2 is essential for human BK virus propagation in tissue culture. J Gen Virol.

[25] McVey D, Brizuela L, Mohr I, Marshak DR, Gluzman Y, Beach D (1989). Phosphorylation of large tumour antigen by cdc2 stimulates SV40 DNA replication. Nature.

[26] Mohr IJ, Stillman B, Gluzman Y (1987). Regulation of SV40 DNA replication by phosphorylation of T antigen. Embo j.

[27] Diaz J, Wang X, Tsang SH, Jiao J, You J (2014). Phosphorylation of large T antigen regulates merkel cell polyomavirus replication. Cancers (Basel).

[28] Poulin DL, Kung AL, DeCaprio JA (2004). p53 targets simian virus 40 large T antigen for acetylation by CBP. J Virol.

[29] Shimazu T, Komatsu Y, Nakayama KI, Fukazawa H, Horinouchi S, Yoshida M (2006). Regulation of SV40 large T-antigen stability by reversible acetylation. Oncogene.

[30] Balakrishnan L, Milavetz B (2008). HDAC inhibitors stimulate viral transcription by multiple mechanisms. Virol J.

[31] Wollebo HS, Woldemichaele B, Khalili K, Safak M, White MK (2013). Epigenetic regulation of polyomavirus JC. Virol J.

[32] Drazic A, Myklebust LM, Ree R, Arnesen T (2016). The world of protein acetylation. Biochim Biophys Acta.

[33] Narita T, Weinert BT, Choudhary C. Functions and mechanisms of non-histone protein acetylation. Nat Rev Mol Cell Biol 2018. 10.1038/s41580-018-0081-3 30467427

[34] Mirzaei H, Ghorbani S, Khanizadeh S, Namdari H, Faghihloo E, Akbari A (2020). Histone deacetylases in virus-associated cancers. Rev Med Virol.

[35] Narita T, Weinert BT, Choudhary C (2019). Functions and mechanisms of non-histone protein acetylation. Nat Rev Mol Cell Biol.

[36] Olsen JV, Mann M (2013). Status of large-scale analysis of post-translational modifications by mass spectrometry. Mol Cell Proteomics.

[37] Seto E, Yoshida M (2014). Erasers of histone acetylation: the histone deacetylase enzymes. Cold Spring Harb Perspect Biol.

[38] Fujimoto H, Higuchi M, Koike M, Ode H, Pinak M, Bunta JK, Nemoto T, Sakudoh T, Honda N, Maekawa H (2012). A possible overestimation of the effect of acetylation on lysine residues in KQ mutant analysis. J Comput Chem.

[39] Kamieniarz K, Schneider R (2009). Tools to tackle protein acetylation. Chem Biol.

[40] Caron C, Boyault C, Khochbin S (2005). Regulatory cross-talk between lysine acetylation and ubiquitination: role in the control of protein stability. BioEssays.

[41] Grönroos E, Hellman U, Heldin CH, Ericsson J (2002). Control of Smad7 stability by competition between acetylation and ubiquitination. Mol Cell.

[42] Bacon T, Seiler C, Wolny M, Hughes R, Watson P, Schwabe J, Grigg R, Peckham M (2015). Histone deacetylase 3 indirectly modulates tubulin acetylation. Biochem J.

[43] Han KA, Kang HS, Lee JW, Yoo L, Im E, Hong A, Lee YJ, Shin WH, Chung KC (2014). Histone deacetylase 3 promotes RCAN1 stability and nuclear translocation. PLoS ONE.

