# Supplementary material for: Histone deacetylase III interactions with BK polyomavirus large tumor antigen may affect protein stability
Source: Virol J. 2023 Jul 18;20:155. doi: 10.1186/s12985-023-02128-6 (PMC10354968; doi:10.1186/s12985-023-02128-6)
Supplement: Supplementary file 1 — Supplementary Material 1 [file 12985_2023_2128_MOESM1_ESM.docx]

Histone deacetylase III interactions with BK polyomavirus Large Tumor Antigen and may affect protein stability

**Fig. S1** Mass spectrometric analysis of BKPyV LT protein acetylation. (A) Flow chart of mass spectrometry. (B) Acetylation modification spectrum of K3 (C) and K230 (D) was shown.

**Construction of K3R, K230R, and K3R/K230R mutation BKPyV LT plasmid**

I. BKPyV LT: Wild-type LT

(A) Wild-type LT nucleotide sequence

ATGGAC**AAG**GTGCTGAATAGAGAAGAGAGTATGGAACTGATGGATCTGCTGGGGCTGGAACGGGCTGCCTGGGGAAATCTGCCACTGATGAGGAAGGCCTACCTGCGCAAGTGCAAGGAGTTCCACCCCGACAAGGGCGGCGACGAGGATAAGATGAAGAGGATGAACACACTGTATAAGAAGATGGAGCAGGACGTGAAGGTGGCCCACCAGCCAGATTTCGGCACCTGGAGCTCCTCTGAGGTGCCCACCTACGGCACAGAGGAGTGGGAGAGCTGGTGGAGCTCCTTTAATGAGAAGTGGGACGAGGATCTGTTCTGTCACGAGGACATGTTTGCCTCCGATGAGGAGGCCACCGCCGACTCTCAGCACAGCACACCCCCCAAGAAGAAGCGGAAGGTGGAGGACCCAAAGGATTTCCCCTCCGATCTGCACCAGTTCCTGAGCCAGGCCGTGTTTTCCAACAGAACCCTGGCCTGCTTTGCCGTGTACACCACAAAGGAGAAGGCCCAGATCCTGTACAAGAAGCTGATGGAGAAGTATTCTGTGACATTCATCAGCAGGCACATGTGCGCCGGCCACAATATCATCTTCTTTCTGACCCCACACAGGCACCGCGTGAGCGCCATCAACAATTTTTGCCAGAAGCTGTGCACATTCTCCTTTCTGATCTGCAAGGGCGTGAAC**AAG**GAGTACCTGCTGTATTCTGCCCTGACCCGCGACCCCTACCACACAATCGAGGAGAGCATCCAGGGCGGCCTGAAGGAGCACGATTTCAATCCCGAGGAGCCTGAGGAGACAAAGCAGGTGAGCTGGAAGCTGATCACCGAGTATGCCGTGGAGACAAAGTGCGAGGACGTGTTCCTGCTGCTGGGCATGTACCTGGAGTTTCAGTATAACGTGGAGGAGTGCAAGAAGTGTCAGAAGAAGGATCAGCCTTACCACTTTAAGTATCACGAGAAGCACTTCGCCAACGCCATCATCTTTGCCGAGTCCAAGAATCAGAAGTCTATCTGTCAGCAGGCCGTGGACACAGTGCTGGCCAAGAAGCGGGTGGATACCCTGCACATGACAAGGGAGGAGATGCTGACCGAGCGGTTCAACCACATCCTGGACAAGATGGATCTGATCTTTGGAGCACACGGAAATGCCGTGCTGGAGCAGTACATGGCAGGAGTGGCATGGCTGCACTGCCTGCTGCCAAAGATGGACTCTGTGATCTTCGATTTTCTGCACTGTATCGTGTTCAATGTGCCCAAGCGGAGATATTGGCTGTTTAAGGGCCCTATCGACAGCGGCAAGACCACACTGGCAGCAGGCCTGCTGGATCTGTGCGGAGGCAAGGCCCTGAACGTGAATCTGCCTATGGAGAGACTGACATTCGAGCTGGGCGTGGCCATCGACCAGTACATGGTGGTGTTTGAGGATGTGAAGGGAACCGGAGCAGAGAGCAAGGACCTGCCATCCGGCCACGGCATCAACAATCTGGATTCCCTGAGGGACTATCTGGATGGCTCTGTGAAGGTGAACCTGGAGAAGAAGCACCTGAATAAGCGCACCCAGATTTTCCCACCCGGCCTGGTGACAATGAACGAGTACCCTGTGCCAAAGACCCTGCAGGCCCGGTTCGTGAGACAGATCGACTTTCGGCCCAAAATCTACCTGAGAAAGTCCCTGCAGAACAGCGAGTTTCTGCTGGAGAAGAGGATCCTGCAGTCCGGCATGACACTGCTGCTGCTGCTGATCTGGTTCCGCCCTGTGGCCGACTTTGCCACCGATATCCAGTCCCGGATCGTGGAGTGGAAGGAGAGACTGGACTCCGAGATCAGCATGTACACCTTCTCTCGGATGAAGTATAACATCTGCATGGGCAAGTGTATCCTGGATATCACCAGAGAGGAGGACTCTGAGACAGAGGATAGCGGCCACGGCTCTAGCACAGAGAGCCAGTCCCAGTGTTCCTCTCAGGTGAGCGACACCTCCGCCCCTGCAGAGGACTCTCAGCGGAGCGATCCACACAGCCAGGAGCTGCATCTGTGCAAAGGGTTCCAGTGCTTCAAACGACCAAAAACTCCTCCTCCAAAAGAATTCTGCAGATATCCAGCACAGTGGCGGCCGCTCGAGTCTAGAGGGCCCGAGCAGAAACTCATCTCTGAAGAGGATCTGTGATAAACCCGCTGATCAGCCTCGACTGTGCCTTCTAGTTGCCAGCCATCT

(B) Wild-type LT protein sequence

MD**K**VLNREESMELMDLLGLERAAWGNLPLMRKAYLRKCKEFHPDKGGDEDKMKRMNTLYK

KMEQDVKVAHQPDFGTWSSSEVPTYGTEEWESWWSSFNEKWDEDLFCHEDMFASDEEATA

DSQHSTPPKKKRKVEDPKDFPSDLHQFLSQAVFSNRTLACFAVYTTKEKAQILYKKLMEK

YSVTFISRHMCAGHNIIFFLTPHRHRVSAINNFCQKLCTFSFLICKGVN**K**EYLLYSALTR

DPYHTIEESIQGGLKEHDFNPEEPEETKQVSWKLITEYAVETKCEDVFLLLGMYLEFQYN

VEECKKCQKKDQPYHFKYHEKHFANAIIFAESKNQKSICQQAVDTVLAKKRVDTLHMTRE

EMLTERFNHILDKMDLIFGAHGNAVLEQYMAGVAWLHCLLPKMDSVIFDFLHCIVFNVPK

RRYWLFKGPIDSGKTTLAAGLLDLCGGKALNVNLPMERLTFELGVAIDQYMVVFEDVKGT

GAESKDLPSGHGINNLDSLRDYLDGSVKVNLEKKHLNKRTQIFPPGLVTMNEYPVPKTLQ

ARFVRQIDFRPKIYLRKSLQNSEFLLEKRILQSGMTLLLLLIWFRPVADFATDIQSRIVE

WKERLDSEISMYTFSRMKYNICMGKCILDITREEDSETEDSGHGSSTESQSQCSSQVSDT

SAPAEDSQRSDPHSQELHLCKGFQCFKRPKTPPPKEFCRYPAQWRPLESRGPEQKLISEE

DL**TR*SASTVPSSCQPS

II. BKPyV LT: K3R_LT

(A) K3R_LT nucleotide sequence

ATGGAC**CGG**GTGCTGAATAGAGAAGAGAGTATGGAACTGATGGATCTGCTGGGGCTGGAACGGGCTGCCTGGGGAAATCTGCCACTGATGAGGAAGGCCTACCTGCGCAAGTGCAAGGAGTTCCACCCCGACAAGGGCGGCGACGAGGATAAGATGAAGAGGATGAACACACTGTATAAGAAGATGGAGCAGGACGTGAAGGTGGCCCACCAGCCAGATTTCGGCACCTGGAGCTCCTCTGAGGTGCCCACCTACGGCACAGAGGAGTGGGAGAGCTGGTGGAGCTCCTTTAATGAGAAGTGGGACGAGGATCTGTTCTGTCACGAGGACATGTTTGCCTCCGATGAGGAGGCCACCGCCGACTCTCAGCACAGCACACCCCCCAAGAAGAAGCGGAAGGTGGAGGACCCAAAGGATTTCCCCTCCGATCTGCACCAGTTCCTGAGCCAGGCCGTGTTTTCCAACAGAACCCTGGCCTGCTTTGCCGTGTACACCACAAAGGAGAAGGCCCAGATCCTGTACAAGAAGCTGATGGAGAAGTATTCTGTGACATTCATCAGCAGGCACATGTGCGCCGGCCACAATATCATCTTCTTTCTGACCCCACACAGGCACCGCGTGAGCGCCATCAACAATTTTTGCCAGAAGCTGTGCACATTCTCCTTTCTGATCTGCAAGGGCGTGAAC**AAG**GAGTACCTGCTGTATTCTGCCCTGACCCGCGACCCCTACCACACAATCGAGGAGAGCATCCAGGGCGGCCTGAAGGAGCACGATTTCAATCCCGAGGAGCCTGAGGAGACAAAGCAGGTGAGCTGGAAGCTGATCACCGAGTATGCCGTGGAGACAAAGTGCGAGGACGTGTTCCTGCTGCTGGGCATGTACCTGGAGTTTCAGTATAACGTGGAGGAGTGCAAGAAGTGTCAGAAGAAGGATCAGCCTTACCACTTTAAGTATCACGAGAAGCACTTCGCCAACGCCATCATCTTTGCCGAGTCCAAGAATCAGAAGTCTATCTGTCAGCAGGCCGTGGACACAGTGCTGGCCAAGAAGCGGGTGGATACCCTGCACATGACAAGGGAGGAGATGCTGACCGAGCGGTTCAACCACATCCTGGACAAGATGGATCTGATCTTTGGAGCACACGGAAATGCCGTGCTGGAGCAGTACATGGCAGGAGTGGCATGGCTGCACTGCCTGCTGCCAAAGATGGACTCTGTGATCTTCGATTTTCTGCACTGTATCGTGTTCAATGTGCCCAAGCGGAGATATTGGCTGTTTAAGGGCCCTATCGACAGCGGCAAGACCACACTGGCAGCAGGCCTGCTGGATCTGTGCGGAGGCAAGGCCCTGAACGTGAATCTGCCTATGGAGAGACTGACATTCGAGCTGGGCGTGGCCATCGACCAGTACATGGTGGTGTTTGAGGATGTGAAGGGAACCGGAGCAGAGAGCAAGGACCTGCCATCCGGCCACGGCATCAACAATCTGGATTCCCTGAGGGACTATCTGGATGGCTCTGTGAAGGTGAACCTGGAGAAGAAGCACCTGAATAAGCGCACCCAGATTTTCCCACCCGGCCTGGTGACAATGAACGAGTACCCTGTGCCAAAGACCCTGCAGGCCCGGTTCGTGAGACAGATCGACTTTCGGCCCAAAATCTACCTGAGAAAGTCCCTGCAGAACAGCGAGTTTCTGCTGGAGAAGAGGATCCTGCAGTCCGGCATGACACTGCTGCTGCTGCTGATCTGGTTCCGCCCTGTGGCCGACTTTGCCACCGATATCCAGTCCCGGATCGTGGAGTGGAAGGAGAGACTGGACTCCGAGATCAGCATGTACACCTTCTCTCGGATGAAGTATAACATCTGCATGGGCAAGTGTATCCTGGATATCACCAGAGAGGAGGACTCTGAGACAGAGGATAGCGGCCACGGCTCTAGCACAGAGAGCCAGTCCCAGTGTTCCTCTCAGGTGAGCGACACCTCCGCCCCTGCAGAGGACTCTCAGCGGAGCGATCCACACAGCCAGGAGCTGCATCTGTGCAAAGGGTTCCAGTGCTTCAAACGACCAAAAACTCCTCCTCCAAAAGAATTCTGCAGATATCCAGCACAGTGGCGGCCGCTCGAGTCTAGAGGGCCCGAGCAGAAACTCATCTCTGAAGAGGATCTGTGATAAACCCGCTGATCAGCCTCGACTGTGCCTTCT

(B) K3R_LT protein sequence

MD**R**VLNREESMELMDLLGLERAAWGNLPLMRKAYLRKCKEFHPDKGGDEDKMKRMNTLYK

KMEQDVKVAHQPDFGTWSSSEVPTYGTEEWESWWSSFNEKWDEDLFCHEDMFASDEEATA

DSQHSTPPKKKRKVEDPKDFPSDLHQFLSQAVFSNRTLACFAVYTTKEKAQILYKKLMEK

YSVTFISRHMCAGHNIIFFLTPHRHRVSAINNFCQKLCTFSFLICKGVN**K**EYLLYSALTR

DPYHTIEESIQGGLKEHDFNPEEPEETKQVSWKLITEYAVETKCEDVFLLLGMYLEFQYN

VEECKKCQKKDQPYHFKYHEKHFANAIIFAESKNQKSICQQAVDTVLAKKRVDTLHMTRE

EMLTERFNHILDKMDLIFGAHGNAVLEQYMAGVAWLHCLLPKMDSVIFDFLHCIVFNVPK

RRYWLFKGPIDSGKTTLAAGLLDLCGGKALNVNLPMERLTFELGVAIDQYMVVFEDVKGT

GAESKDLPSGHGINNLDSLRDYLDGSVKVNLEKKHLNKRTQIFPPGLVTMNEYPVPKTLQ

ARFVRQIDFRPKIYLRKSLQNSEFLLEKRILQSGMTLLLLLIWFRPVADFATDIQSRIVE

WKERLDSEISMYTFSRMKYNICMGKCILDITREEDSETEDSGHGSSTESQSQCSSQVSDT

SAPAEDSQRSDPHSQELHLCKGFQCFKRPKTPPPKEFCRYPAQWRPLESRGPEQKLISEE

DL**TR*SASTVPS

III. BKPyV LT: K230R LT

(A) K230R_LT nucleotide sequence

ATGGAC**AAG**GTGCTGAATAGAGAAGAGAGTATGGAACTGATGGATCTGCTGGGGCTGGAACGGGCTGCCTGGGGAAATCTGCCACTGATGAGGAAGGCCTACCTGCGCAAGTGCAAGGAGTTCCACCCCGACAAGGGCGGCGACGAGGATAAGATGAAGAGGATGAACACACTGTATAAGAAGATGGAGCAGGACGTGAAGGTGGCCCACCAGCCAGATTTCGGCACCTGGAGCTCCTCTGAGGTGCCCACCTACGGCACAGAGGAGTGGGAGAGCTGGTGGAGCTCCTTTAATGAGAAGTGGGACGAGGATCTGTTCTGTCACGAGGACATGTTTGCCTCCGATGAGGAGGCCACCGCCGACTCTCAGCACAGCACACCCCCCAAGAAGAAGCGGAAGGTGGAGGACCCAAAGGATTTCCCCTCCGATCTGCACCAGTTCCTGAGCCAGGCCGTGTTTTCCAACAGAACCCTGGCCTGCTTTGCCGTGTACACCACAAAGGAGAAGGCCCAGATCCTGTACAAGAAGCTGATGGAGAAGTATTCTGTGACATTCATCAGCAGGCACATGTGCGCCGGCCACAATATCATCTTCTTTCTGACCCCACACAGGCACCGCGTGAGCGCCATCAACAATTTTTGCCAGAAGCTGTGCACATTCTCCTTTCTGATCTGCAAGGGCGTGAAC**CGG**GAGTACCTGCTGTATTCTGCCCTGACCCGCGACCCCTACCACACAATCGAGGAGAGCATCCAGGGCGGCCTGAAGGAGCACGATTTCAATCCCGAGGAGCCTGAGGAGACAAAGCAGGTGAGCTGGAAGCTGATCACCGAGTATGCCGTGGAGACAAAGTGCGAGGACGTGTTCCTGCTGCTGGGCATGTACCTGGAGTTTCAGTATAACGTGGAGGAGTGCAAGAAGTGTCAGAAGAAGGATCAGCCTTACCACTTTAAGTATCACGAGAAGCACTTCGCCAACGCCATCATCTTTGCCGAGTCCAAGAATCAGAAGTCTATCTGTCAGCAGGCCGTGGACACAGTGCTGGCCAAGAAGCGGGTGGATACCCTGCACATGACAAGGGAGGAGATGCTGACCGAGCGGTTCAACCACATCCTGGACAAGATGGATCTGATCTTTGGAGCACACGGAAATGCCGTGCTGGAGCAGTACATGGCAGGAGTGGCATGGCTGCACTGCCTGCTGCCAAAGATGGACTCTGTGATCTTCGATTTTCTGCACTGTATCGTGTTCAATGTGCCCAAGCGGAGATATTGGCTGTTTAAGGGCCCTATCGACAGCGGCAAGACCACACTGGCAGCAGGCCTGCTGGATCTGTGCGGAGGCAAGGCCCTGAACGTGAATCTGCCTATGGAGAGACTGACATTCGAGCTGGGCGTGGCCATCGACCAGTACATGGTGGTGTTTGAGGATGTGAAGGGAACCGGAGCAGAGAGCAAGGACCTGCCATCCGGCCACGGCATCAACAATCTGGATTCCCTGAGGGACTATCTGGATGGCTCTGTGAAGGTGAACCTGGAGAAGAAGCACCTGAATAAGCGCACCCAGATTTTCCCACCCGGCCTGGTGACAATGAACGAGTACCCTGTGCCAAAGACCCTGCAGGCCCGGTTCGTGAGACAGATCGACTTTCGGCCCAAAATCTACCTGAGAAAGTCCCTGCAGAACAGCGAGTTTCTGCTGGAGAAGAGGATCCTGCAGTCCGGCATGACACTGCTGCTGCTGCTGATCTGGTTCCGCCCTGTGGCCGACTTTGCCACCGATATCCAGTCCCGGATCGTGGAGTGGAAGGAGAGACTGGACTCCGAGATCAGCATGTACACCTTCTCTCGGATGAAGTATAACATCTGCATGGGCAAGTGTATCCTGGATATCACCAGAGAGGAGGACTCTGAGACAGAGGATAGCGGCCACGGCTCTAGCACAGAGAGCCAGTCCCAGTGTTCCTCTCAGGTGAGCGACACCTCCGCCCCTGCAGAGGACTCTCAGCGGAGCGATCCACACAGCCAGGAGCTGCATCTGTGCAAAGGGTTCCAGTGCTTCAAACGACCAAAAACTCCTCCTCCAAAAGAATTCTGCAGATATCCAGCACAGTGGCGGCCGCTCGAGTCTAGAGGGCCCGAGCAGAAACTCATCTCTGAAGAGGATCTGTGATAAACCCGCTGATCAGCCTCGACTGTGCCTTC

(B) K230R_LT protein sequence

MD**K**VLNREESMELMDLLGLERAAWGNLPLMRKAYLRKCKEFHPDKGGDEDKMKRMNTLYK

KMEQDVKVAHQPDFGTWSSSEVPTYGTEEWESWWSSFNEKWDEDLFCHEDMFASDEEATA

DSQHSTPPKKKRKVEDPKDFPSDLHQFLSQAVFSNRTLACFAVYTTKEKAQILYKKLMEK

YSVTFISRHMCAGHNIIFFLTPHRHRVSAINNFCQKLCTFSFLICKGVN**R**EYLLYSALTR

DPYHTIEESIQGGLKEHDFNPEEPEETKQVSWKLITEYAVETKCEDVFLLLGMYLEFQYN

VEECKKCQKKDQPYHFKYHEKHFANAIIFAESKNQKSICQQAVDTVLAKKRVDTLHMTRE

EMLTERFNHILDKMDLIFGAHGNAVLEQYMAGVAWLHCLLPKMDSVIFDFLHCIVFNVPK

RRYWLFKGPIDSGKTTLAAGLLDLCGGKALNVNLPMERLTFELGVAIDQYMVVFEDVKGT

GAESKDLPSGHGINNLDSLRDYLDGSVKVNLEKKHLNKRTQIFPPGLVTMNEYPVPKTLQ

ARFVRQIDFRPKIYLRKSLQNSEFLLEKRILQSGMTLLLLLIWFRPVADFATDIQSRIVE

WKERLDSEISMYTFSRMKYNICMGKCILDITREEDSETEDSGHGSSTESQSQCSSQVSDT

SAPAEDSQRSDPHSQELHLCKGFQCFKRPKTPPPKEFCRYPAQWRPLESRGPEQKLISEE

DL**TR*SASTVPS

IV. BKPyV LT: K3R/K230R LT

(A) K3R/K230R_LT nucleotide sequence

ATGGAC**CGG**GTGCTGAATAGAGAAGAGAGTATGGAACTGATGGATCTGCTGGGGCTGGAACGGGCTGCCTGGGGAAATCTGCCACTGATGAGGAAGGCCTACCTGCGCAAGTGCAAGGAGTTCCACCCCGACAAGGGCGGCGACGAGGATAAGATGAAGAGGATGAACACACTGTATAAGAAGATGGAGCAGGACGTGAAGGTGGCCCACCAGCCAGATTTCGGCACCTGGAGCTCCTCTGAGGTGCCCACCTACGGCACAGAGGAGTGGGAGAGCTGGTGGAGCTCCTTTAATGAGAAGTGGGACGAGGATCTGTTCTGTCACGAGGACATGTTTGCCTCCGATGAGGAGGCCACCGCCGACTCTCAGCACAGCACACCCCCCAAGAAGAAGCGGAAGGTGGAGGACCCAAAGGATTTCCCCTCCGATCTGCACCAGTTCCTGAGCCAGGCCGTGTTTTCCAACAGAACCCTGGCCTGCTTTGCCGTGTACACCACAAAGGAGAAGGCCCAGATCCTGTACAAGAAGCTGATGGAGAAGTATTCTGTGACATTCATCAGCAGGCACATGTGCGCCGGCCACAATATCATCTTCTTTCTGACCCCACACAGGCACCGCGTGAGCGCCATCAACAATTTTTGCCAGAAGCTGTGCACATTCTCCTTTCTGATCTGCAAGGGCGTGAAC**CGG**GAGTACCTGCTGTATTCTGCCCTGACCCGCGACCCCTACCACACAATCGAGGAGAGCATCCAGGGCGGCCTGAAGGAGCACGATTTCAATCCCGAGGAGCCTGAGGAGACAAAGCAGGTGAGCTGGAAGCTGATCACCGAGTATGCCGTGGAGAGCATCCAGGGCGGCCTGAAGGAGCACGATTTCAATCCCGAGGAGCCTGAGGAGACAAAGCAGGTGAGCTGGAAGCTGATCACCGAGTATGCCGTGGAGACAAAGTGCGAGGACGTGTTCCTGCTGCTGGGCATGTACCTGGAGTTTCAGTATAACGTGGAGGAGTGCAAGAAGTGTCAGAAGAAGGATCAGCCTTACCACTTTAAGTATCACGAGAAGCACTTCGCCAACGCCATCATCTTTGCCGAGTCCAAGAATCAGAAGTCTATCTGTCAGCAGGCCGTGGACACAGTGCTGGCCAAGAAGCGGGTGGATACCCTGCACATGACAAGGGAGGAGATGCTGACCGAGCGGTTCAACCACATCCTGGACAAGATGGATCTGATCTTTGGAGCACACGGAAATGCCGTGCTGGAGCAGTACATGGCAGGAGTGGCATGGCTGCACTGCCTGCTGCCAAAGATGGACTCTGTGATCTTCGATTTTCTGCACTGTATCGTGTTCAATGTGCCCAAGCGGAGATATTGGCTGTTTAAGGGCCCTATCGACAGCGGCAAGACCACACTGGCAGCAGGCCTGCTGGATCTGTGCGGAGGCAAGGCCCTGAACGTGAATCTGCCTATGGAGAGACTGACATTCGAGCTGGGCGTGGCCATCGACCAGTACATGGTGGTGTTTGAGGATGTGAAGGGAACCGGAGCAGAGAGCAAGGACCTGCCATCCGGCCACGGCATCAACAATCTGGATTCCCTGAGGGACTATCTGGATGGCTCTGTGAAGGTGAACCTGGAGAAGAAGCACCTGAATAAGCGCACCCAGATTTTCCCACCCGGCCTGGTGACAATGAACGAGTACCCTGTGCCAAAGACCCTGCAGGCCCGGTTCGTGAGACAGATCGACTTTCGGCCCAAAATCTACCTGAGAAAGTCCCTGCAGAACAGCGAGTTTCTGCTGGAGAAGAGGATCCTGCAGTCCGGCATGACACTGCTGCTGCTGCTGATCTGGTTCCGCCCTGTGGCCGACTTTGCCACCGATATCCAGTCCCGGATCGTGGAGTGGAAGGAGAGACTGGACTCCGAGATCAGCATGTACACCTTCTCTCGGATGAAGTATAACATCTGCATGGGCAAGTGTATCCTGGATATCACCAGAGAGGAGGACTCTGAGACAGAGGATAGCGGCCACGGCTCTAGCACAGAGAGCCAGTCCCAGTGTTCCTCTCAGGTGAGCGACACCTCCGCCCCTGCAGAGGACTCTCAGCGGAGCGATCCACACAGCCAGGAGCTGCATCTGTGCAAAGGGTTCCAGTGCTTCAAACGACCAAAAACTCCTCCTCCAAAAGAATTCTGCAGATATCCAGCACAGTGGCGGCCGCTCGAGTCTAGAGGGCCCGAGCAGAAACTCATCTCTGAAGAGGATCTGTGATAAACCCGCTGATCAGCCTCGACTGTGCCTTCTAG

(B) K3R/K230R_protein sequence

MD**R**VLNREESMELMDLLGLERAAWGNLPLMRKAYLRKCKEFHPDKGGDEDKMKRMNTLYK

KMEQDVKVAHQPDFGTWSSSEVPTYGTEEWESWWSSFNEKWDEDLFCHEDMFASDEEATA

DSQHSTPPKKKRKVEDPKDFPSDLHQFLSQAVFSNRTLACFAVYTTKEKAQILYKKLMEK

YSVTFISRHMCAGHNIIFFLTPHRHRVSAINNFCQKLCTFSFLICKGVN**R**EYLLYSALTR

DPYHTIEESIQGGLKEHDFNPEEPEETKQVSWKLITEYAVESIQGGLKEHDFNPEEPEET

KQVSWKLITEYAVETKCEDVFLLLGMYLEFQYNVEECKKCQKKDQPYHFKYHEKHFANAI

IFAESKNQKSICQQAVDTVLAKKRVDTLHMTREEMLTERFNHILDKMDLIFGAHGNAVLE

QYMAGVAWLHCLLPKMDSVIFDFLHCIVFNVPKRRYWLFKGPIDSGKTTLAAGLLDLCGG

KALNVNLPMERLTFELGVAIDQYMVVFEDVKGTGAESKDLPSGHGINNLDSLRDYLDGSV

KVNLEKKHLNKRTQIFPPGLVTMNEYPVPKTLQARFVRQIDFRPKIYLRKSLQNSEFLLE

KRILQSGMTLLLLLIWFRPVADFATDIQSRIVEWKERLDSEISMYTFSRMKYNICMGKCI

LDITREEDSETEDSGHGSSTESQSQCSSQVSDTSAPAEDSQRSDPHSQELHLCKGFQCFK

RPKTPPPKEFCRYPAQWRPLESRGPEQKLISEEDL**TR*SASTVPSX

**Fig. S2** The nucleotide (A) and amino acids (B) sequence of wild-type (I), K3R (II), K230R (III), and K3R/K230R (IV), BKPyV LT. The K3 and K230 residues are presented in red.

**siHDAC1**


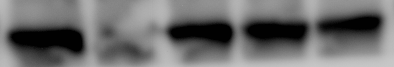

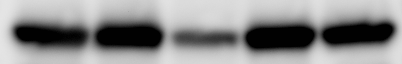

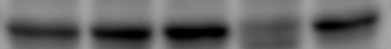


**HDAC1**

**HDAC2**

**HDAC3**

**HDAC8**

**Mock**

**siHDAC2**

**siHDAC3**

**siHDAC8**


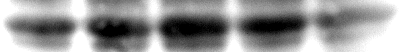


**Fig. S3** HDACs protein expression after siRNA treatment. Specific siRNA to HDACs were transfected to HK-2 cells. HDACs expressions were determined by western blotting after 48 hours of transfection.

Table S1 siRNA sequence and primer pairs used in this study.

| Target gene | Sense | Antisense |
| --- | --- | --- |
| siHDAC1(s73) | CUAUGGUCUCUACCGAAAATT | UUUUCGGUAGAGACCAUAGTT |
| siHDAC1(s75) | GGGUUGUUUCAAUCUAACATT | UGUUAGAUUGAAACAACCCAG |
| siHDAC2 (s6493) | GGGUUGUUUCAAUCUAACATT | UGUUAGAUUGAAACAACCCAG |
| siHDAC2(s6494) | GGCAGAUAUUUAAGCCUAUTT | AUAGGCUUAAAUAUCUGCCCA |
| siHDAC3(s16878) | GGAGCUUCCCUAUAGUGAATT | UUCACUAUAGGGAAGCUCCTC |
| siHDAC3(s16876) | GAGCUUCAAUAUCCCUCUATT | UAGAGGGAUAUUGAAGCUCTT |
| siHDAC8((s31698) | GGUCCCGGUUUAUAUCUAUTT | AUAGAUAUAAACCGGGACCAG |
| siHDAC8((s31697) | GGACGGUACUACAGUGUAATT | UUACACUGUAGUACCGUCCCT |
| K3R | CAAAAATGGATCGCGTTCTTAACAGGGAAGAATCCATG | CATGGATTCTTCCCTGTTAAGAACGCGATCCATTTTTG |
| K230R | TAATTTGTAAGGGTGTTAATCGCGAATACTTACTATATAG | CTATATAGTAAGTATTCGCGATTAACACCCTTACAAATTA |
